# Pyrene Excimer-Based Fluorescent Labeling of Cysteines Brought into Close Proximity by Protein Dynamics: ASEM-Induced Thiol-Ene Click Reaction for High Spatial Resolution CLEM

**DOI:** 10.3390/ijms21207550

**Published:** 2020-10-13

**Authors:** Masami Naya, Chikara Sato

**Affiliations:** 1 Health and Medical Research Institute, National Institute of Advanced Industrial Science and Technology (AIST), Tsukuba 305-8566, Japan; m.naya@aist.go.jp; 2 Master’s and Doctoral Programs in Neuroscience, Graduate School of Comprehensive Human Sciences, University of Tsukuba, Tsukuba 305-8574, Japan

**Keywords:** excimer fluorescence, chemical reaction, correlative light and electron microscopy (CLEM), atmospheric scanning electron microscopy (ASEM), post modification, cysteine scanning mutagenesis, protein misfolding diseases, cancer radiotherapy, electron beam lithography

## Abstract

Fluorescence microscopy (FM) has revealed vital molecular mechanisms of life. Mainly, molecules labeled by fluorescent probes are imaged. However, the diversity of labeling probes and their functions remain limited. We synthesized a pyrene-based fluorescent probe targeting SH groups, which are important for protein folding and oxidative stress sensing in cells. The labeling achieved employs thiol-ene click reactions between the probes and SH groups and is triggered by irradiation by UV light or an electron beam. When two tagged pyrene groups were close enough to be excited as a dimer (excimer), they showed red-shifted fluorescence; theoretically, the proximity of two SH residues within ~30 Å can thus be monitored. Moreover, correlative light/electron microscopy (CLEM) was achieved using our atmospheric scanning electron microscope (ASEM); radicals formed in liquid by the electron beam caused the thiol-ene click reactions, and excimer fluorescence of the labeled proteins in cells and tissues was visualized by FM. Since the fluorescent labeling is induced by a narrow electron beam, high spatial resolution labeling is expected. The method can be widely applied to biological fields, for example, to study protein dynamics with or without cysteine mutagenesis, and to beam-induced micro-fabrication and the precise post-modification of materials.

## 1. Introduction

Fluorescence microscopy (FM) visualizes various phenomena in many research fields. The super-resolution microscopies [1,2,3,4] exceed the resolution limit of diffraction limited FM and provide excellent images of live cells. The fluorescent probes required by most FM have been developed and adapted to visualize various aspects of cells, such as green fluorescent protein (GFP), cameleon using fluorescence resonance energy transfer (FRET), many kinds of calcium indicators, and so on [5,6,7]. At the same time, high-resolution electron microscopy (EM) has evolved. The fine structures of biological samples and materials have been imaged by transmission electron microscopy (TEM) or scanning electron microscopy (SEM). The short wavelength of electrons has achieved high-resolution scanning of samples with a very narrow electron beam. Scanning transmission electron microscopy (STEM) fully exploits this advantage and has revealed the high-resolution structures of various materials; a resolution as high as 0.405 Å was achieved for a gallium nitride (GaN) crystal [8]. Cryo-TEM and -STEM allow for the observation of biological samples in hydrophilic conditions [9], and three-dimensional structures have been determined at high resolution [10]. Furthermore, liquid-phase EM has been developed. An environmental capsule with electron-permeable thin windows accommodates wet samples and allows them to be observed using TEM or STEM [11,12]. We developed atmospheric scanning electron microscopy (ASEM), which is able to image wet samples in containers that are open to atmosphere at high resolution [13]. Not only biological samples including bacteria [14] and cancer cells or tissues [15], but also crystal growth [16] and complex metal precipitation processes forming delicate nanonetworks [17] have been observed using ASEM.

FM and EM have their own characteristics and yield different types of the information; FM observes targets labeled by differently colored fluorescence, while EM observes targets with single wavelength electrons at high resolution and results in greyscale images of the sample. Correlative light-electron microscopy (CLEM) approaches successfully combine the benefits of FM and EM [18,19,20,21,22,23]. Various hybrid probes have been developed for CLEM, including quantum dots (Qdots) and FluoroNanoGold [24,25,26,27,28,29,30,31,32,33,34]. Most recently, cryo-EM techniques have enabled high-resolution CLEM of frozen materials [35,36,37,38,39]. In microscopy techniques, especially CLEM, labeling is the key. However, the diversity of labeling probes and their functions are still limited.

Today, pyrene is used as fluorescent probe for biological FM [40,41,42]. When two pyrene molecules are excited as a dimer, called an excimer, they have red-shifted fluorescence. The fluorescence properties of pyrene excimer are used as a probe, targeting bio-molecules, and can extract spatial information [43,44,45,46,47,48]. For example, pyrene was conjugated with DNA to detect antisense nucleic acids [44]. Furthermore, it was used as a chemosensor for ATP, metal ions, and pH [42,49,50,51].

**Figure 1:**
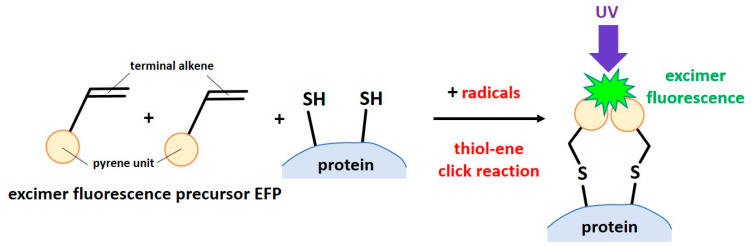
Schematic illustration of thiol-ene click reactions between the excimer fluorescence precursor (**EFP**) and SH groups of a protein. The **EFP** labeling of the neighboring SH groups produces the complex which exhibits pyrene excimer fluorescence.

In this paper, we report the development of a brand new pyrene-based fluorescent labeling of SH groups (Figure 1) to widen the limited targets of pyrene. The fluorescence precursor compound, called excimer fluorescence precursor (**EFP**), was synthesized by conjugating pyrene with alkene to realize thiol-ene click reactions of high reactivity under mild conditions [52,53,54,55] that are applicable for the biological labeling of SH groups. The labeling reaction between the terminal alkene of **EFP** and an SH group of a cysteine residue of protein is caused by a free radical, which can be generated by UV light or an electron beam. When two bound **EFP**s are close, they display excimer fluorescence (Figure 1 right). Closely located SH groups of reduced cells and tissues were labeled with **EFP** and imaged by the excimer fluorescence.

## 2. Results

### 2.1. Synthesis and Characterization of Excimer Fluorescence Precursor (**EFP**)

**Scheme 1:**
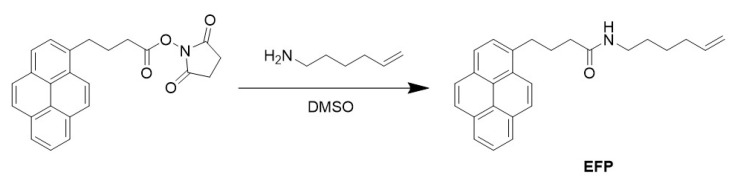
Synthesis of excimer fluorescence precursor **EFP**.

**Figure 2:**
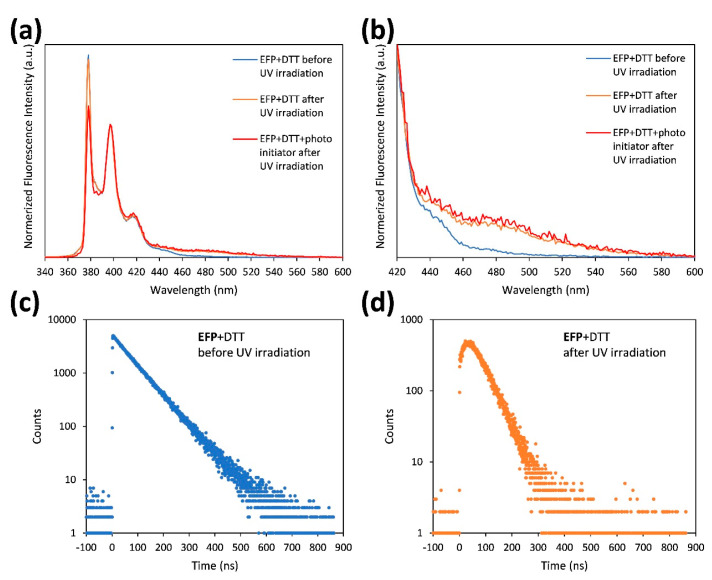
Fluorescence spectra and fluorescence lifetime measurements of **EFP** + DTT before UV irradiation (blue), **EFP** + DTT after UV irradiation (orange), and **EFP** + DTT + photoinitiator after UV irradiation (red) in DMSO. The fluorescence was excited at 342 nm. **EFP** concentration in the reaction mixture was 2.71 × 10^−2^ M and was diluted to the concentration of 8.67 × 10^−6^ M for fluorescence measurements. (**a**) Spectra between 340 and 600 nm. (**b**) Spectra between 420 and 600 nm displayed on a larger scale. After UV irradiation, the spectra showed a broad fluorescence peak in the range of 460–560 nm, which was deduced to derive from pyrene excimers. Fluorescence lifetime measurement of (**c**) **EFP** + DTT (blue dots; measured at 378 nm) and (**d**) **EFP** + DTT after UV irradiation (orange dots; measured at 479 nm) in DMSO. The time-lapse measurement in (**d**) exhibited slow peak rise of the excimer fluorescence, suggesting the dynamic mechanism of excimer formation.

The excimer fluorescence precursor (**EFP**) was synthesized, as indicated in Scheme 1, and characterized by ^1^H NMR and HRMS (high resolution mass spectrometry). Because the pyrene unit is hydrophobic and the amide group is hydrophilic, the synthesized **EFP** was not dissolved in pure water, but in a dimethyl sulfoxide (DMSO)/H_2_O 9:1 (*v*/*v*) mixture; it was not soluble in water alone. The optical properties of **EFP** and a model **EFP** dimer obtained by reacting **EFP** with dithiothreitol (DTT) in the solution (Scheme S1), were characterized (Figure S1, UV absorption spectroscopy; Figure 2, fluorescence spectroscopy). The **EFP** dimer was prepared as follows: A solution of **EFP** and DTT in DMSO was irradiated by UV light with or without 1-hydroxycyclohexyl phenyl ketone (photoinitiator). As shown in Figure 2a,b, **EFP** had no fluorescence above 500 nm, while the **EFP** dimer showed a broad peak. Because DTT has two SH groups per molecule, the **EFP** dimer obtained tended to form a pyrene excimer. The thiol-ene click reactions between **EFP**s and DTT were probably triggered by free radicals of DTT SH groups. The photoinitiator was not effective, presumably due to some side reactions, and there was no significant increase in the conversion of the reaction (Table S1).

Next, fluorescence lifetime measurements were conducted. As shown in Figure 2c,d, the **EFP** dimer solution exhibited slow peak rise (Figure 2d) while unirradiated **EFP** + DTT exhibited linear excitation and decay curves (Figure 2c). These results suggest that the pyrene units of an **EFP** dimer are excited as monomers at first, and complexed to a dimer, and then emit the excimer fluorescence.

### 2.2. **EFP** Labeling of COS-7 Cells Using UV Light

**Figure 3:**
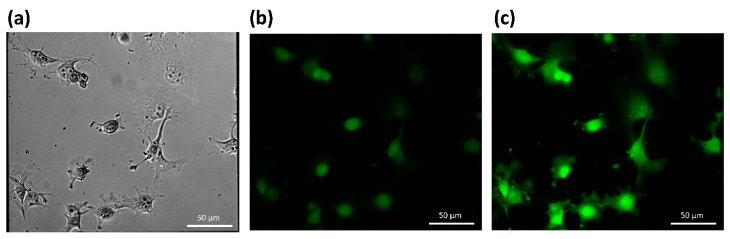
Excimer fluorescence of COS-7 cells before and after the **EFP** labeling induced by UV irradiation. Cells were fixed and reduced in advance. (**a**) Phase contrast microscopic image of COS-7 cells. (**b**) Fluorescence microscopy (FM) image of COS-7 cells before the **EFP** labeling. (**c**) FM image of COS-7 cells labeled with **EFP** using thiol-ene click reaction triggered by UV irradiation. Cells were brighter after the labeling; the nuclei were intensively labeled.

**Figure 4:**
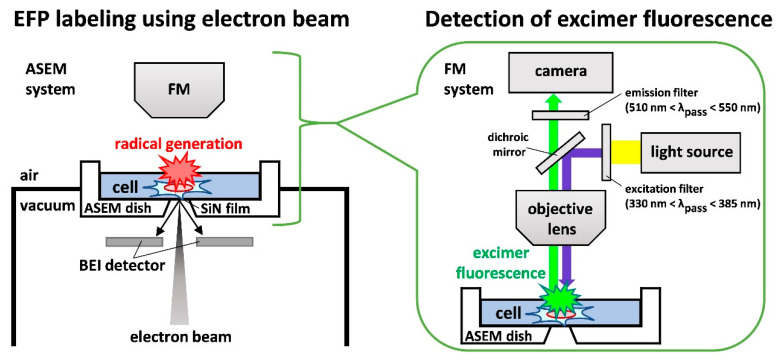
The atmospheric scanning electron microscope (ASEM) system for the **EFP** labeling and detection of pyrene excimer fluorescence of neighboring SH residues. The electron beam from the inverted SEM at the bottom was used to induce the thiol-ene click reaction (left), and the FM system at the top was used to visualize the excimer fluorescence (right). The electron-transparent silicon nitride (SiN) film windows [15] of the ASEM dish separates the atmosphere and the vacuum in the column [13].

To demonstrate the **EFP** labeling of the cells, reduced COS-7 cells were labeled by UV light and observed using FM. The cultured COS-7 cells were fixed by 4% paraformaldehyde (PFA), and washed by PBS buffer (Figure 3a). The cells had a slight autofluorescence in the nuclei before the labeling (Figure 3b). SH groups of proteins in the cells were reduced by DTT, and the solvent was replaced by DMSO/H_2_O 9:1 solution containing **EFP** (10 mg/mL); then the cells were irradiated by UV light without permeabilization, because both **EFP** and DMSO are hydrophobic. Afterwards, the cells were washed with PBS buffer, and the pyrene excimer fluorescence of the sample was observed using FM (Figure 4 right). The irradiated cells were brightly imaged (Figure 3c); the cytoplasm was moderately bright while nuclei, and especially nucleoli, were brighter. These results suggest that the thiol-ene click reaction between the reduced proteins of the cells and **EFP** was triggered by the UV irradiation, and that **EFP** protein complexes with the excimer fluorescence were formed between the neighboring free SH groups of the reduced cells. Based on the image intensities, there are more neighboring SH groups in the nuclei, especially in the nucleoli, than in the cytoplasm.

### 2.3. CLEM of COS-7 Cells Using **EFP** Labeling and ASEM

In order to assess the use of **EFP** labeling for CLEM, COS-7 cells were labeled using the electron beam of an ASEM to induce the required free radicals and imaged in situ from above by FM (Figure 4). The ASEM had an inverted SEM. A wet sample immersed in liquid was placed on a disposable ASEM dish with electron-transparent silicon nitride (SiN) film windows in its base. The base of the dish sealed the column of the inverted SEM and the windows allowed the sample to be scanned at atmospheric pressure from below by the electron beam. ASEM visualized backscattered electrons (BE) from the sample using a BE imaging (BEI) detector, and at the same time the irradiated molecules, especially water molecules, turned into free radicals for the thiol-ene click reaction (Figure 4 left) [56].

**Figure 5:**
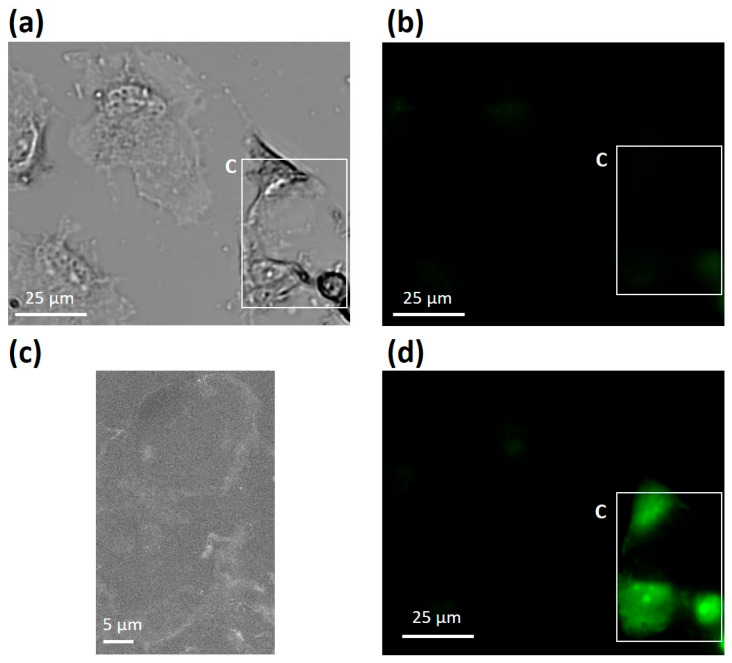
Fluorescence of fixed and reduced COS-7 cells before and after electron beam-induced **EFP** labeling for a new correlative light-electron microscopy (CLEM). Cells were fixed and reduced in advance. (**a**) Phase contrast microscopy image and (**b**) FM image of the cells before region C was scanned by the electron beam. (**c**) SEM image of the cells present within region C in (**b**). (**d**) FM image of the cells after they were scanned by the electron beam of the inverted SEM. The cells scanned by the electron beam in area C were imaged brighter than the surroundings using FM.

For the experiment, COS-7 cells were cultured in the ASEM dish in advance. The cells were fixed using 4% PFA, without permeabilization, reduced by DTT, and inspected by FM to image the autofluorescence (Figure 5a,b). The cells were then immersed in DMSO/H_2_O 9:1 and stained with 2% phosphotungstic acid (PTA) to make them clearly visible using the inverted SEM. The stain solution was replaced by DMSO/H_2_O 9:1 solution containing **EFP** (10 mg/mL), and only area C of Figure 5 was irradiated and imaged by ASEM (Figure 5c). The cells were then washed with DMSO/H_2_O 9:1 and observed using the FM of the ASEM. Only the cells in area C emitted strong excimer fluorescence (Figure 5d), while the image of cells that were not irradiated remained faint. These results suggest that electron beam scanning generated radicals, the radicals triggered the thiol-ene click reaction between **EFP** and proteins, and then the excimer fluorescence was induced. The radicals were deduced to be mainly free radicals of SH groups and water molecules. **EFP** labeling together with narrow electron beam scanning suggests that high spatial resolution labeling of biological cells could become a new CLEM.

### 2.4. CLEM of Mouse Hippocampal Tissue Using **EFP** Labeling and ASEM

**Figure 6:**
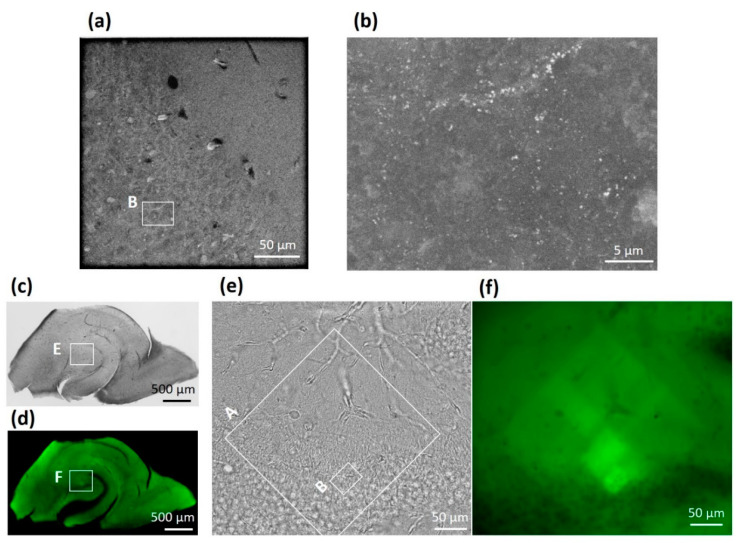
Mouse hippocampal tissue imaged using electron beam-induced **EFP** labeling and CLEM. The tissue was aldehyde-fixed, reduced, PCG/PTA stained, and **EFP**-labeled using the electron beam of an ASEM. (**a**) Low magnification SEM image of the tissue in a window; the outermost black border is the window frame. (**b**) Higher magnification SEM image of area B in (**a**). Precise structures of the hippocampus, including networks, were imaged by the inverted SEM. (**c**) Phase contrast microscopy and (**d**) FM of the tissue after the electron beam scanning. (**e**) Higher magnification phase contrast image of rectangle E indicated in (**c**). Areas A and B in (**e**) correspond to the scanned areas (SEM images) shown in (**a**,**b**). (**f**) Higher magnification FM image of the white rectangle F indicated in (**d**). The square area scanned at low magnification by the inverted SEM (i.e., the region shown in (**a**)) was imaged brighter than the surroundings, and the rectangular area scanned at higher magnification (i.e., the region shown in (**b**)) was imaged much brighter.

To extend CLEM using **EFP** to biological tissue samples, mouse brain tissue was **EFP**-labeled, and the excimer fluorescence was imaged using ASEM. A perfusion-fixed (4% PFA) hippocampus of adult mouse was sectioned to 100 µm thickness slabs and further fixed with 2.5% glutaraldehyde (GA). The tissue was stained for EM by positively charged nanogold (PCG), without permeabilization, and reduced by DTT. The solvent was then replaced with DMSO/H_2_O 9:1, and the tissue was further stained by PTA. After the stain solution was replaced by DMSO/H_2_O 9:1 solution containing **EFP** (10 mg/mL), the tissue was observed by the inverted SEM (Figure 6a,b). Fine structures and networks in the tissue were imaged through the windows of the ASEM dish. The tissue was washed with DMSO/H_2_O 9:1, and observed by FM (Figure 6c–f). Although the tissue had relatively high autofluorescence (Figure 6d), the rectangular areas scanned by the electron beam were clearly brighter than the surroundings, which were not scanned; one of the rectangles matched the square shape of an ASEM window (bright square in Figure 6f, which corresponds to area A in Figure 6e, and further to Figure 6a in which the surrounding silicon window frame is imaged dark at the edge). Furthermore, ASEM observation at higher magnification (Figure 6b) provided a much brighter rectangle in the FM image (bottom of the bright square in Figure 6f, which corresponds to area B in Figure 6e). These results suggest that the **EFP** labeling of tissues using the thiol-ene click reaction was induced by electron beam scanning, and that neighboring cysteine residues exhibited the excimer fluorescence.

## 3. Discussion

In this article, we report a brand new fluorescent labeling method employing thiol-ene click reactions between **EFPs** and free SH groups. Characterization and conversions of the **EFP** labeled model compound with or without a photoinitiator were estimated by ^1^H NMR. The results suggest that the **EFP** labeling was mainly caused by free radicals of SH groups. Therefore, the **EFP** chemoselectively labels SH groups under relatively mild conditions.

As an example of cell labeling, reduced COS-7 cells were **EFP**-labeled using UV irradiation and imaged by FM (Figure 3). From the fact that the excimer fluorescence of the irradiated cells was higher than that of the unirradiated cells, it was suggests that free radicals induced by UV irradiation triggered thiol-ene click reactions and the neighboring pyrene residues formed excimers.

The **EFP** labeling probably targets free SH groups of proteins, and two labeled cysteines must be close to form an excimer. The longest distance between SH groups that can result in the excimer fluorescence can be estimated. The distance between the pyrene groups of the excimer cannot be clearly defined in solution; as the distance changes, fluorescence intensity of the excimer alters. However, to obtain visible excimer fluorescence, the distance between the two pyrene rings is required to be within 3–5 Å [45,46]. Considering the linker length and the high flexibility of **EFP**, it is assumed that the longest distance between the two SH residues is within 28–30 Å.

In this study, the pretreatment of samples included the reduction of disulfide bonds formed between the cysteines of proteins using DTT. Therefore, FM of the **EFP** labeled cells might have mainly visualized the distribution of the reduced disulfide bonds, and the contribution of SH groups that are accidentally in close proximity to one another might be minor. Because disulfide bonds play an important role in protein folding and function, this labeling method has the potential to be applied to study protein folding and protein misfolding diseases, such as prion diseases and Alzheimer’s disease. Moreover, because **EFP** labeling of non-reduced cells theoretically only tags free SH groups that have accidentally become neighbors, the observed fluorescence might represent the redox state of proteins in the cell. If the image of such free SH groups is subtracted from the image of all the labeled cysteine residues of reduced samples, the residual image may represent the distribution of disulfide bonds. Because free SH groups are known as oxidative stress sensors in cells, this analysis of free SH could be useful to study cancers, lifestyle diseases, and aging, which are all related to oxidative stresses. Furthermore, the combination of the **EFP** labeling and cysteine scanning mutagenesis [57,58] has the potential to provide an important methodology to study protein structure, dynamics, and mechanisms, including catalytic activities of enzymes, redox sensing, signaling, and the function of motor proteins.

Electron beam scanning was able to activate the **EFP** labeling in cells and tissues, and the excimer fluorescence was imaged by FM as a new CLEM (Figure 5 and Figure 6). This electron beam-induced **EFP** labeling observed in the scanned square could be developed further as a fine spot labeling, because the diameter of the electron beam of a standard SEM is less than 10 nm. Moreover, the flow from EM to FM presented here is the reverse of the order usually employed in CLEM. These characteristics suggest the possibility of electron beam-induced **EFP** labeling combined with simultaneous FM, creating a new approach for super-resolution microscopy. Because this **EFP** labeling can be combined with STEM, which has a very narrow electron beam (<1 Å in diameter [8,12,59]), it is expected to realize high spatial resolution labeling microscopy and might be applied for imaging and microprocessing.

For further development, it is possible to change the emission wavelength of **EFP** excimer using chemical modification or another fluorophore: Multi-color excimer imaging is expected to visualize the detailed function of proteins. A new molecular design of **EFP** including different kinds of hydrophilic groups including poly(ethylene glycol) chain could improve the solubility of **EFP** in water.

This novel fluorescent labeling has the potential to be widely applied in various studies, e.g., in super-resolution microscopy, in basic biology, and in X-ray radiotherapy of cancer cells [60,61], by exploiting the X-ray induced radicals, and also to radiotherapy using electrons, electron beam lithography for fine processing technologies and nanomaterials constructions, and to achieve the precise post modification of materials. In the future, this labeling might be further developed as an important research and clinical probe for the diagnoses of diseases due to high spatial resolution fluorescence labeling.

## 4. Materials and Methods

### 4.1. Materials and Instruments

Unless noted otherwise, all reagents were obtained from commercial sources and used without further purification. The 1-pyrenebutyric acid *N*-hydroxysuccinimide ester was obtained from Tokyo Chemical Industry Co., Ltd. (Tokyo, Japan). Dimethyl sulfoxide was obtained from Fujifilm Wako Pure Chemical Corporation (Osaka, Japan). DMSO-*d*_6_, was obtained from Sigma-Aldrich Inc. (St. Louis, MO, USA). UV absorption spectroscopy was performed using a DU-800 spectrophotometer (Beckman-Coulter, Brea, CA, USA). Fluorescence spectra were measured using a LS50B fluorescence spectrometer (Perkin Elmer, Waltham, MA, USA). Time-resolved fluorescence measurement was performed using a single photon counter FluoroCube (Horiba, Kyoto, Japan). ^1^H NMR spectra were recorded using an Avance III-500 NMR instrument (Bruker, Billerica, MA) at the Biomedical Research Institute, National Institute of Advanced Industrial Science and Technology (AIST). HRMS was performed by the Open Research Facilities Station, TIA Central Office, AIST.

### 4.2. Synthesis of Excimer Fluorescence Precursor **EFP**

**Scheme 2:**
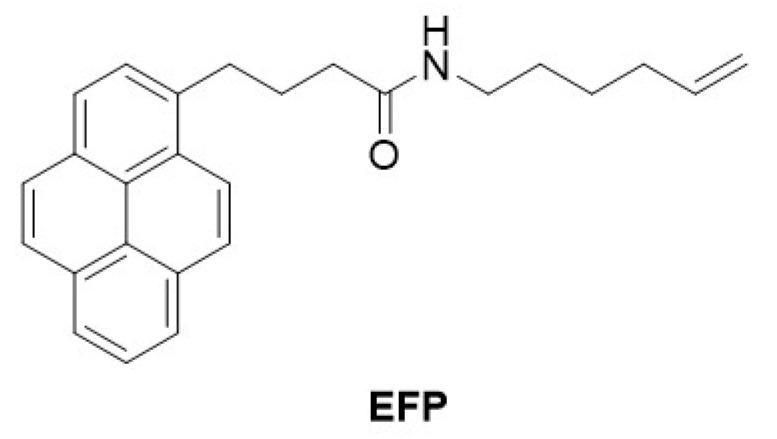
*N*-(hex-5-en-1-yl)-4-(pyren-1-yl)butanamide (**EFP**): White solid. ^1^H NMR (500 MHz, DMSO-*d*_6_, r.t.): *δ* (ppm) 7.91–8.41 (m, 9*H*, pyrene), 7.83 (s, 1*H*, NH), 5.72–5.84 (m, 1*H*, alkene), 4.99 (d, *J* = 17.2, 1*H*, alkene), 4.92 (d, *J* = 9.45, 1*H*, alkene), 3.31 (t, *J* = 7.8, 2H), 3.24 (q, *J* = 6.1, 2H), 2.22 (t, *J* = 7.1, 2*H*), 1.97–2.06 (m, 4*H*), 1.31–1.45 (m, 4*H*). HRMS (EI) *m*/*z*: M^+^ calc’d for C_26_H_27_NO 369.2093, found: 369.2091.

To a solution of 5-hexene-1-amine (51.5 mg, 0.519 mmol) in DMSO (10 mL), 1-pyrenebutyric acid *N*-hydroxysuccinimide ester (200 mg, 0.519 mmol) was added. The mixture was stirred overnight at room temperature and then poured into double distilled water (DDW). The mixture was filtrated. The precipitation on the filter was washed with DDW and dried under vacuum to obtain the purified compound (Scheme 2) as a white solid (112 mg, 0.302 mmol, 58%).

### 4.3. Thiol-Ene Click Reaction between **EFP** and DTT

A solution of **EFP** (10 mg/mL) and DTT (8.7 mg/mL) with or without 1-hydroxycyclohexyl phenyl ketone (6.0 mg/mL) in DMSO or DMSO-*d*_6_ (200 µL) was irradiated by a UV transilluminator (Funakoshi, TM-40, 15 W, 254 nm) for 30 min. The solution after the reaction was diluted to 3125 times, and UV absorption spectroscopy (Figure S1) and fluorescence spectra (Figure 2) were recorded (excited at 342 nm). ^1^H NMR spectroscopy was performed for the solution after the reaction, and the conversion was estimated (Table S1).

### 4.4. Thiol-Ene Click Reaction Between **EFP** and COS-7 Cells

COS-7 cells were cultured in Dulbecco’s modified Eagle’s medium (DMEM) supplemented with 10% fetal bovine serum (FBS) and 100 μg/mL kanamycin, in a 5% CO_2_ atmosphere at 37 °C in the ASEM dish, fixed by 4% paraformaldehyde, and reduced in DTT (1 mM) aqueous solution for 30 min without permeabilization, because both **EFP** and DMSO are hydrophobic. Afterwards, the liquid was replaced by a DMSO/H_2_O 9:1 (*v*/*v*) mixture. The sample was washed by repeatedly removing and replacing the DMSO/H_2_O 9:1 solution, and stained by 2% phosphotungstic acid (PTA) in DMSO/H_2_O 9:1 solution for 30 min. Afterwards, the sample was again washed with DMSO/H_2_O 9:1 solution, and the solvent was replaced by DMSO/H_2_O 9:1 solution containing **EFP** (10 mg/mL). Then, the cells were irradiated through the SiN film windows at the bottom of the ASEM dish by the UV transilluminator for 30 min or by the electron beam of the inverted SEM (30 kV), which was scanned over the region at a magnification of 370× for 40 min. They were then washed with DMSO/H_2_O 9:1 and observed from above by FM.

### 4.5. Animals

Four week-old male Jcl:ICR mice (Japan Clea, Tokyo, Japan) were sacrificed to obtain tissues for observation. The animal studies were in compliance with the national institute rules of conduct and adhered to the principles of Institutional Animal Care and Use Committee Guidebook. All experiments were approved (DOU2019-0048, 30 July 2019) by the Animal Care and Use Committee of the National Institute of Advanced Industrial Science and Technology (AIST).

### 4.6. Tissue Sample Preparation

Animals were anesthetized using isoflurane (Abbott, Maidenhead, UK) and sacrificed by intracardiac perfusion of 4% paraformaldehyde (PFA; Wako Pure Chemicals, Osaka, Japan) in phosphate-buffered saline (PBS, pH 7.4). Tissues were sliced with a PRO7 linear slicer (Dosaka, Kyoto, Japan) to obtain 100 μm thick slabs. Samples were washed several times in PBS and further fixed with 2.5% glutaraldehyde (GA) (Nisshin EM, Tokyo, Japan) for 30 min at RT; the fixative volume was 15–20 times the sample volume.

### 4.7. Thiol-Ene Click Reaction between **EFP** and Adult Mouse Hippocampal Tissue

After sectioning of a hippocampus of adult mouse to 100 µm thickness, the hippocampal slab was further fixed with 2.5% glutaraldehyde (GA). Without permeabilization, the tissue was labeled by positively charged nanogold^®^ (PCG) and Goldenhance^TM^ EM, as described in [33], and reduced in 1 mM DTT aqueous solution for 30 min. Then, the solvent was replaced by a DMSO/H_2_O 9:1 mixture, and the tissue was stained with PTA for 30 min. After, the solvent was replaced by a DMSO/H_2_O 9:1 solution containing **EFP** (10 mg/mL), and the inverted SEM (30 kV) was used to observe the sample for 54 min in the longest case. The sample was then washed with DMSO/H_2_O 9:1, and observed from above by FM.

### 4.8. Microscopy

ASEM images were recorded using the ClairScope ASEM system (JASM-6200), JEOL, Ltd., Tokyo, Japan. ASEM dishes with eight windows [14] were employed. The brain tissue slab was sectioned, fixed, and stained as described, and placed on the SiN film windows of the ASEM dish. The sample was immediately imaged by optical microscopy and SEM [13]. The electron dose at the highest magnification of 6000× was 8.3 e^−^/Å^2^, which is 17.7% of the dose of 47 e^−^/Å^2^ permitted in low-dose cryo-electron microscopy aiming at atomic resolution single particle reconstructions. FM and optical microscopy was performed using an Olympus BX-50 with a high-pressure mercury lamp (USH-102D, 100 W), a dichroic mirror (DM400; 400 nm < λ transmitted), a bandpass excitation filter (BP330-385; 330 nm< λ < 385 nm transmitted), a bandpass emission filter (BP510-550; 510 nm < λ < 550 nm transmitted), and 10×, 40× or 100× objective lenses (NA:0.30, 0.60, or 1.00, respectively), which was an integral part of the ASEM set-up.

## 5. Conclusions

In this paper, we report the development of a new pyrene-based excimer fluorescence labeling. The labeling employed a thiol-ene click reaction between biological samples and the excimer fluorescence precursor **EFP**, and SH groups of the proteins in reduced cells and tissues were labeled using radicals generated by UV light or an electron beam. As a CLEM, the ASEM/FM observation of cells or tissues was demonstrated; the labeling reaction was induced by the electron beam of an inverted SEM, and the excimer fluorescence of the samples was observed by FM. The labeling was restricted to the area scanned by the electron beam, which suggests the possibility of the space-limited labeling within a narrow electron beam trajectory leading to high resolution. It should be exploited as a new method to visualize neighboring SH groups by spot-scanning cells or tissues. This method could provide a great advantage for biological analytics of oxidative stress sensing and protein folding. By labeling the free SH groups of a protein with the pyrene derivatives, the excimer fluorescence could report the dynamic motility of the protein as a proximity sensor. Overall, **EFP**-based CLEM and protein dynamics studies might play a crucial role for studies of diseases and clinical applications including X-ray radiodynamic therapy for cancer. This labeling method has the potential to be widely applied for various science fields including biology, nanomaterial chemistry, and molecular physics.

## Supplementary Materials

Supplementary materials can be found at https://www.mdpi.com/1422-0067/21/20/7550/s1. Scheme S1: Thiol-ene click reaction between **EFP** and DTT. Figure S1: UV absorption spectra of **EFP**+DTT before and after UV irradiation. Table S1: Conversion of the thiol-ene click reaction between **EFP** and DTT.

## Abbreviations

ASEM	Atmospheric SEM
DMSO	Dimethyl sulfoxide
DTT	Dithiothreitol
EFP	Excimer fluorescence precursor
EM	Electron microscopy/microscope
FM	Fluorescence microscopy/microscope
FRET	Fluorescence resonance energy transfer
GA	Glutaraldehyde
GFP	Green fluorescent protein
PCG	Positively charged gold^®^
PFA	Paraformaldehyde
PTA	Phosphotungstic acid
SEM	Scanning EM
STEM	Scanning transmission EM
TEM	Transmission EM
