# Supplementary material for: Pyrene Excimer-Based Fluorescent Labeling of Cysteines Brought into Close Proximity by Protein Dynamics: ASEM-Induced Thiol-Ene Click Reaction for High Spatial Resolution CLEM
Source: Int J Mol Sci. 2020 Oct 13;21(20):7550. doi: 10.3390/ijms21207550 (PMC7589919; doi:10.3390/ijms21207550)
Supplement: Supplementary file 1 [file ijms-21-07550-s001.zip › ijms-938855-supplementary.docx]

*Supporting Information for*

Pyrene Excimer-Based Fluorescent Labeling of Cysteines Neighbored by Protein Dynamics: ASEM-induced Thiol-Ene Click Reaction for High Spatial Resolution CLEM

Masami Naya ^1^ and Chikara Sato ^1,2^

1. Health and Medical Research Institute, National Institute of Advanced Industrial Science and Technology (AIST), Tsukuba 305-8566, Japan; m.naya@aist.go.jp
2. Master’s and Doctoral Programs in Neuroscience, Graduate School of Comprehensive Human Sciences, University of Tsukuba, Tsukuba 305-8574, Japan; ti-sato@aist.go.jp

1. Evaluation of Thiol-ene Click Reaction between EFP and Dithiothreitol (DTT) and Their Optical Properties

The thiol-ene click reaction between **EFP** and DTT (Scheme S1) was performed in DMSO or DMSO-*d*_6_ by UV irradiation for 30 min with or without 1-hydroxycyclohexyl phenyl ketone (photoinitiator). The optical properties of the samples were evaluated before and after the reaction, by UV spectroscopy (Figure S1), fluorescence spectroscopy (Figure 2a,b) and time-resolved fluorescence spectroscopy (Figure 2c,d). The conversion was estimated by ^1^H NMR (Table S1).


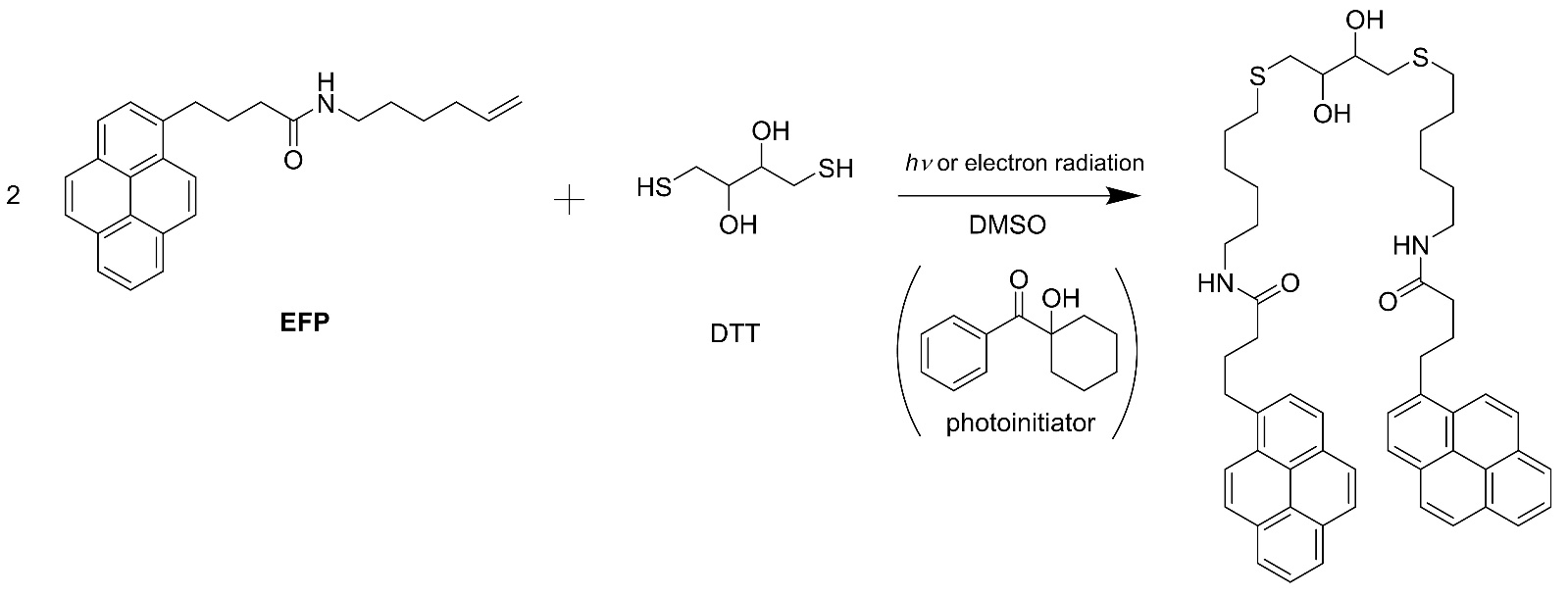


**Scheme S1.** Thiol-ene click reaction between **EFP** and dithiothreitol (DTT) induced by UV irradiation. The reaction product was evaluated as a model for complexes formed between of the **EFP** and proteins.


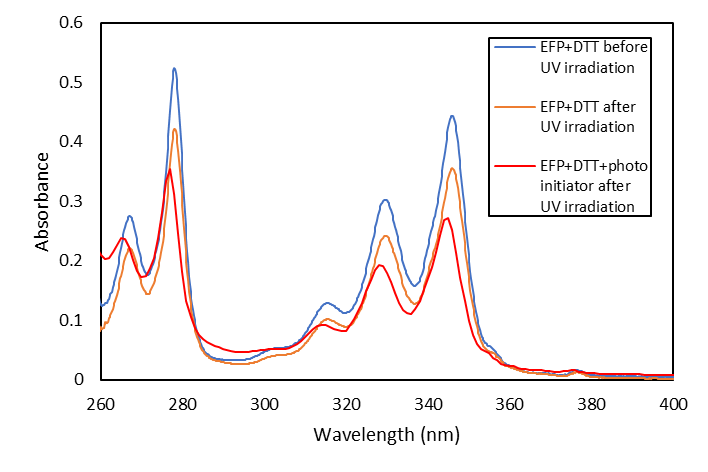


**Figure S1.** UV absorption spectra of **EFP**+DTT before UV irradiation (blue), **EFP** + DTT after UV irradiation (orange) and **EFP** + DTT + photoinitiator after UV irradiation (red) in DMSO solution.

**Table S1.** Conversion of the thiol-ene click reaction between **EFP** and DTT with or without photoinitiator induced by UV irradiation.

|  | **Conversion ^a^** |
| --- | --- |
| With photoinitiator | 31.6% |
| Without photoinitiator | 43.7% |

a Conversion was estimated by the ratio of the ^1^H NMR integral of alkenyl proton and pyrenyl proton.
